# Clinical and translational medicine: Integrative and practical science. 

**DOI:** 10.1186/2001-1326-1-1

**Published:** 2012-03-29

**Authors:** Edward Abraham, Francesco M Marincola, Zhinan Chen, Xiangdong Wang

**Affiliations:** 1 Wake Forest School of Medicine, Medical Center Boulevard, Winston-Salem, NC 27157, USA; 2 Infectious Disease and Immunogenetics Section, Department of Transfusion Medicine, Clinical Center, Trans-NIH Center for Human Immunology, National Institutes of Health, Bethesda, USA; 3 Academician of National Academy of Engineering, Cell Engineering Research Center at the Fourth Military Medical University, Xi'an, China; 4 Biomedical Research Center, Fudan University Zhongshan Hospital, Shanghai, China & Clinical Science, Lund University, Lund, Sweden

## 

Clinical and translational medicine plays a unique and critical role in fostering the flow of bidirectional information between basic and clinical scientists, optimizing new biotechnologies, improving clinical application of new therapeutic concepts, and ultimately improving the quality of life for patients. The term "Clinical and Translational Medicine" (CMT) is defined here as "clinical potential and application of translational research and science to improve the understanding of mechanisms and therapies of human diseases", a new and important concept for the development of disease-specific biomarkers and therapeutic strategies to monitor and cure disease. ***Clinical and Translational Medicine ****as *an international open-access journal publish articles focused on novel advances in clinical, translational, and clinically-relevant basic science, to accelerate the transition from preclinical research to clinical applications and the communication between basic and clinical scientists. ***Clinical and Translational Medicine ***is particularly interested in drug discovery and development, related regulatory processes, as well as broader topics related to public health and relevant policies. ***Clinical and Translational Medicine ***will also focus on new insights into molecular mechanisms, clinical questions and experience, as well as the discovery and development of personalized healthcare delivery.

Clinical and translational science has been defined as a novel attempt to "translate remarkable scientific innovations into health gains", and is a critical and core component of full-spectrum biomedical research [1]. Translational research as a key word was emphasized and headlined in the National Institutes of Health guide for Specialized Program of Research Excellence (SPORE) grants for cancer research [2]. Furthermore, translational science has been defined as a two-way process to translate discoveries from the bench into clinical application and/or the translation of clinical findings into the understanding of molecular mechanisms [3,4]. Given the growing impact of scientific knowledge and discoveries on clinical practice, translational medicine was initially described as "the marriage between new discoveries in basic science and clinical practice" [5]. Clinical and translational medicine can be used to understand the mechanisms of clinical variation between diseases, pathogenesis, biomarkers, and therapies. For example, translational medicine is involved in determining optimal regimens to alleviate symptoms and improve quality of life. The efficacy and safety of drugs selected from pre-clinical animal models can be translated into applicable and therapeutic approaches for clinical trials [6].

Clinical and translational medicine plays a unique and critical role in fostering the flow of bidirectional information between basic and clinical scientists, optimizing new biotechnologies, improving clinical application of new therapeutic concepts, and ultimately improving the quality of life for patients. Clinical and translational medicine integrates clinical research with modern methodologies in systems and computational biology, genomics, proteomics, metabolomics, pharmacomics, transcriptomics, and high-throughput image analysis. It should also foster the implementation of human tissue banking, and the development of bio-banks linked to high quality clinical data bases, for identification of clear phenotypes relevant to stratification of patients receiving standard or experimental therapies [7,8]. Clinical and translational medicine is a limiting factor and accelerator in the process of moving from preclinical discovery and development to clinical application, validating the application of new technologies in patients, and developing new strategies to improve the quality of life for patients. It adds scientific and evidence-based value to the regulations, policies, and/or guidelines for medications, therapies, preventive approaches, and health care delivery. Thus, there is pressing need for a scientific channel and platform like the journal of ***Clinical and Translational Medicine ***to exchange information on the development, standardization, application, and optimization of translational research and science in a manner that will facilitate communication between preclinical and clinical scientists.

Clinical and translational medicine should be furthermore defined and differentiated from the understanding of other "translational" concepts, including translational science, translational research, translational medicine, or clinical and translational science. In contrast to these broader approaches, clinical and translational medicine is expected to concentrate on clinical application-oriented translational science and research to improve the accuracy, efficiency and efficacy of clinical diagnoses, therapies, and determination of prognoses for patients. Clinical and translational medicine will play an important and applicable role in monitoring and managing the misalignment between the growth in research spending and the decrease in translational productivity, as pointed out recently by Elias Zerhouni [9]. There is great need to exchange knowledge and experience relating to the development of new biotechnologies, as well as to the understanding of gene and protein function, cell and organ dysfunction, and pathology related to clinical signs, symptoms, findings, measures, prognosis, and therapeutic effects. Clinical and translational research using multidimensional data from both molecular biology and medicine thus accelerates and shortens the process to translate preclinical knowledge into clinical applications. Therefore, the journal of ***Clinical and Translational Medicine ***has the special mission and responsibility to emphasize the clinical potential and application of new biotechnologies, particularly with respect to regulation and health policy.

The journal of ***Clinical and Translational Medicine ***will stake out the future landscape of clinical and translational research, present advances in biotechnology, facilitate the fast-tracking of research and development, introduce novel diagnostics and therapeutics for clinical use, and explore the challenges and opportunities in the post-genomic and proteomic era to develop new disciplines that reflect additional levels of complexity. The journal will help clarify bioethics at the interface of biotechnologies and clinical applications, the paradigms of academia-industry and public-private models, and will address the demands of maintaining and expanding the biomedical workforce and education programs that attract and retain clinicians and investigators. The Journal will play an important and critical role in enhancing the understanding of disease-specific molecular mechanisms, and will provide information that facilitates the development of predictive, preventive, personalized medicine, likely to improve of patient prognoses.

The term "*Clinical and Translational Medicine*" is defined here as "clinical potential and application of translational research and science to improve the understanding of mechanisms and therapies of human diseases", a new and important concept for the development of disease-specific biomarkers and therapeutic strategies to monitor and cure disease. In addition, the Journal will encourage and foster the establishment of standardization and consensus of biotechnological processes applied to clinical and translational research, to make preclinical and clinical data more comparable and repeatable. One of the challenging issues in translating preclinical data into clinical application is the wide variation between reports due to different protocols and often uncontrolled conditions. ***Clinical and Translational Medicine ***has the responsibility of reducing such misalignment, establishing standardized databases, and developing applicable consensus. The role of clinical and translational science and research is becoming more important than ever, as we attempt to meet the future needs of clinical and translational medicine, to form more global opinion leaders to bridge the gap between understanding basic science and human disease, and to define the content, regulation and policy of clinical and translational medicine. ***Clinical and Translational Medicine ***will provide a forum for the exchange of ideas on translational skills, methodologies, definitions, protocols, regulations and policies for clinical application and improvement of health care.

***Clinical and Translational Medicine ***is also proud to be affiliated with the newly established International Society of Translational Medicine (ISTM) [10] and will be a primary publication site for clinical and translational science and research associated with ISTM. As a non-profit organization, ISTM is a network of clinicians and researchers from all fields of science with an interest in translational medicine. The partnership between the Journal and ISTM will facilitate interdisciplinary research across clinical medicine and translational science. ***Clinical and Translational Medicine ***encourages publishing scientific collaborations between basic scientists and clinical investigators with the requisite skills to design longitudinal studies and deal with regulatory and human-protection complexities. The Journal provides a forum for basic scientists to collaborate with clinical investigators and has the special policy that formally recognizes more than one investigator as principal author on a publication, as has been previously suggested [11,12].

We, as editors of ***Clinical and Translational Medicine***, are delighted to welcome you to this new journal. We thank the scientists who have already agreed to publish in the journal. In initiating the journal, we owe an enormous debt of gratitude to our colleagues for their encouragement, support, comments, suggestions and contributions. With the support of our Associate Editors and Editorial Board Members [13], we believe that ***Clinical and Translational Medicine ***will be well-received by preclinical, translational, and clinical scientists and will provide an important forum to improve the healthcare of humans.
